# Supplementary material for: Developing a Prognostic Gene Panel of Epithelial Ovarian Cancer Patients by a Machine Learning Model
Source: Cancers (Basel). 2019 Feb 25;11(2):270. doi: 10.3390/cancers11020270 (PMC6406249; doi:10.3390/cancers11020270)
Supplement: Supplementary file 1 [file cancers-11-00270-s001.pdf]

cancers

Supplementary Materials: Developing A Prognostic Gene Panel of Epithelial Ovarian Cancer Patients by A Machine Learning Model

Tzu-Pin Lu, Kuan-Ting Kuo, Ching-Hsuan Chen, Ming-Cheng Chang, Hsiu-Ping Lin, Yu-Hao Hu, Ying-Cheng Chiang, Wen-Fang Cheng and Chi-An Chen

Identification of the genes associated with the chemo-response

The raw CEL files of the gene expression microarrays (GSE36133) in the CCLE project [1] were retrieved from the Gene Expression Omnibus (GEO) [2] (Table S1), and the robust multiarray average (RMA) algorithm was utilized to generate the expression level of one probe. The CCLE dataset contained the gene expression profiles examined by the Affymetrix u133plus 2.0 microarray in several tissue types and the efficacy data for 24 drugs. We selected the 25 ovarian cancer cell lines (Table S2) containing the drug efficacy data as the training set to identify the genes associated with the drug efficacy. The 25 ovarian cancer cell lines were classified into three groups based on their sensitivity, which was the activity area provided by the GSE36133 dataset. Furthermore, a quantile normalization algorithm was performed to reduce systematic biases across the different cell lines. For each probe in the 25 ovarian cancer lines, the gene expression value was adjusted to the standard normal distribution. A Kruskal-Wallis test was performed to identify the probes showing significantly different expression levels in the three groups ($p < 0.01$). Based on the official annotation file provided by Affymetrix, the probes that mapped to none and/or multiple genes were excluded in this study in order to remove ambiguity. When multiple probes were annotated to the same gene, their coefficients of variation (CVs) were calculated, and only the probe possessing the largest CV was kept for further analyses.

Development of a prediction model using a genetic algorithm

Initially, we randomly select 10 probes from those significant probes and repeated this procedure to generate 100 combinations for the first generation. For each combination, the support vector machine (SVM) algorithm was utilized to develop a prediction model, and its prediction accuracy was evaluated by using a leave one out cross-validation. Subsequently, we kept the combination showing the highest accuracy in the first generation to the second generation. Two combinations were selected from the first generation according to the probabilities that were obtained by dividing their accuracy values by the total accuracy values of all the combinations to generate other combinations in the second generation. For each pair of the two selected combinations, the crossover process was executed by randomly exchanging the predictors among them. These procedures were repeated until 10 generations were bred, and the model showing the highest accuracy for predicting the paclitaxel response was developed in the last generation. To evaluate the random chance of identifying 10 probes with the same prediction accuracy, a permutation test was performed by randomly selecting 10 predictors from the same probe pool utilized in the GA analysis 100,000 times to generate a null baseline. Lastly, we determined the empirical p-value of the prediction model by comparing its prediction accuracy with the null baseline, that is, by ranking the accuracy values.

Table S1. The quantitative PCR primers of TaqMan probes.

Gene	Full Name	TaqMan® Primer/Probe Set No.
RHGEF26	Rho guanine nucleotide exchange factor 26	Hs00248943_m1
CP	ceruloplasmin (ferroxidase)	Hs00236810_m1
DIO3	deiodinase, iodothyronine, type III	Hs00956431_s1
DPEP2	dipeptidase 2	Hs00902586_m1
EPS15L1	epidermal growth factor receptor pathway substrate 15-like 1	Hs01021135_m1
LIPC	lipase, hepatic	Hs00165106_m1
LRRC32	leucine rich repeat containing 32	Hs00194136_m1
PPT2	palmitoyl-protein thioesterase 2	Hs00607118_m1
PRIM2	primase, DNA, polypeptide 2 (58kDa)	Hs00168726_m1
UBE2O	ubiquitin conjugating enzyme E2O	Hs01078087_m1

Table S2. Distribution of OS and RFS in the patients classified into three groups.

Dataset	Group	Feature	Sample size (OS)	OS > 5 years (proportion)	Feature	Sample size (RFS)	RFS > 2 years (proportion)
TCGA	Low	OS	86	0.302	RFS	66	0.197
TCGA	Medium	OS	70	0.3	RFS	46	0.217
TCGA	High	OS	28	0.5	RFS	19	0.526
GSE9891	Low	OS	83	0.06	RFS	81	0.235
GSE9891	Medium	OS	75	0.013	RFS	74	0.27
GSE9891	High	OS	37	0.108	RFS	37	0.432
NTUH	Low	OS	19	0.263	RFS	19	0.263
NTUH	Medium	OS	58	0.397	RFS	58	0.345
NTUH	High	OS	7	0.714	RFS	7	0.571

TCGA: The Cancer Genome Atlas; OS: overall survival; RFS: recurrent free survival; NTUH: National Taiwan University.

Table S3. Characteristics of the three microarray datasets.

Dataset	Platform	# of Analyzed Samples	Ref
GSE36133	Affymetrix U133plus2.0	25 [a]	[1]
TCGA ovarian [b]	Affyemtrix U133plus2.0	OS: 184/RFS: 131	[3]
GSE9891 [b]	Affyemtrix U133plus2.0	OS: 195/RFS: 192	[4]

TCGA: The Cancer Genome Atlas; OS: overall survival; RFS: Recurrence-free survival; [a] 25 ovarian cancer cell lines had drug efficacy data responding to paclitaxel treatment [b] Only patients receiving paclitaxel treatment were included in this study.

Table S4. The 25 ovarian cancer lines in GSE36133 utilized as the training set.

Accession	Cell Line	Activity Area	Group	Accession	Cell Line	Activity Area	Group
**GSM886853**	A2780	6.5225	High	GSM887466	OC 314	6.3589	High
**GSM886962**	COV318	2.8757	Low	GSM887467	OC 316	6.3818	High
**GSM886965**	COV504	3.7749	Low	GSM887483	OV-90	3.4582	Low
**GSM887000**	EFO-21	3.8972	Low	GSM887484	OVCAR-4	4.5514	Medium
**GSM887001**	EFO-27	3.8972	Low	GSM887485	OVCAR-8	6.193	High
**GSM887008**	ES-2	5.3368	High	GSM887488	OVMANA	2.7268	Low
**GSM887014**	FU-OV-1	4.3524	Medium	GSM887489	OVSAHO	3.0491	Low
**GSM887080**	Hey-A8	5.1752	Medium	GSM887490	OVTOKO	2.0493	Low
**GSM887160**	IGROV1	3.7989	Low	GSM887598	SK-OV-3	5.0501	Medium
**GSM887178**	JHOS-2	4.1841	Medium	GSM887710	TOV-112D	6.2674	High
**GSM887179**	JHOS-4	5.2994	High	GSM887711	TOV-21G	5.0367	Medium
**GSM887290**	MCAS	6.4269	High	GSM887718	TYK-nu	5.115	Medium
**GSM887456**	NIH:OVCAR-3	5.2836	Medium				

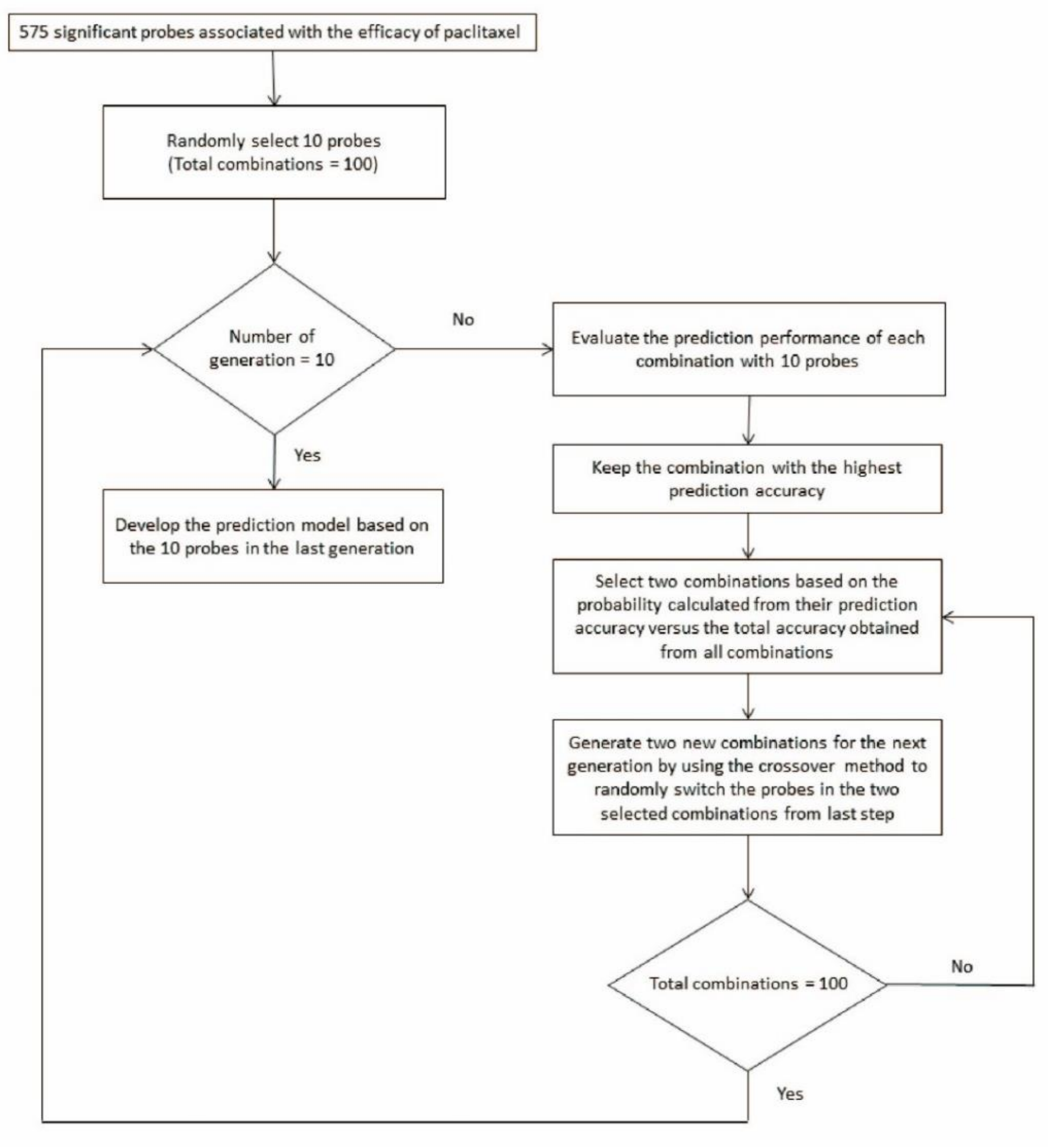

Figure S1. The proposed genetic algorithm (GA) to identify the best combinations of predictors for the SVM model.

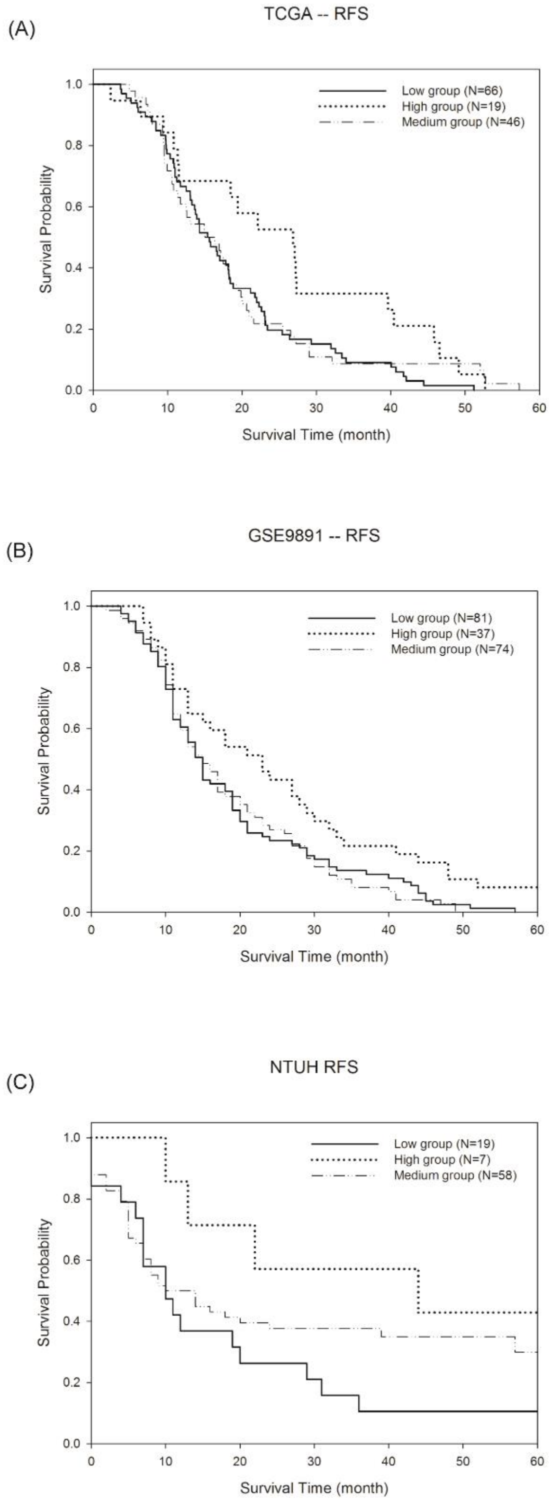

Figure S2. The Kaplan Meier survival curves of the RFS in the three response groups in the (**A**) TCGA, (**B**) GSE9891 and (**C**) NTUH datasets.

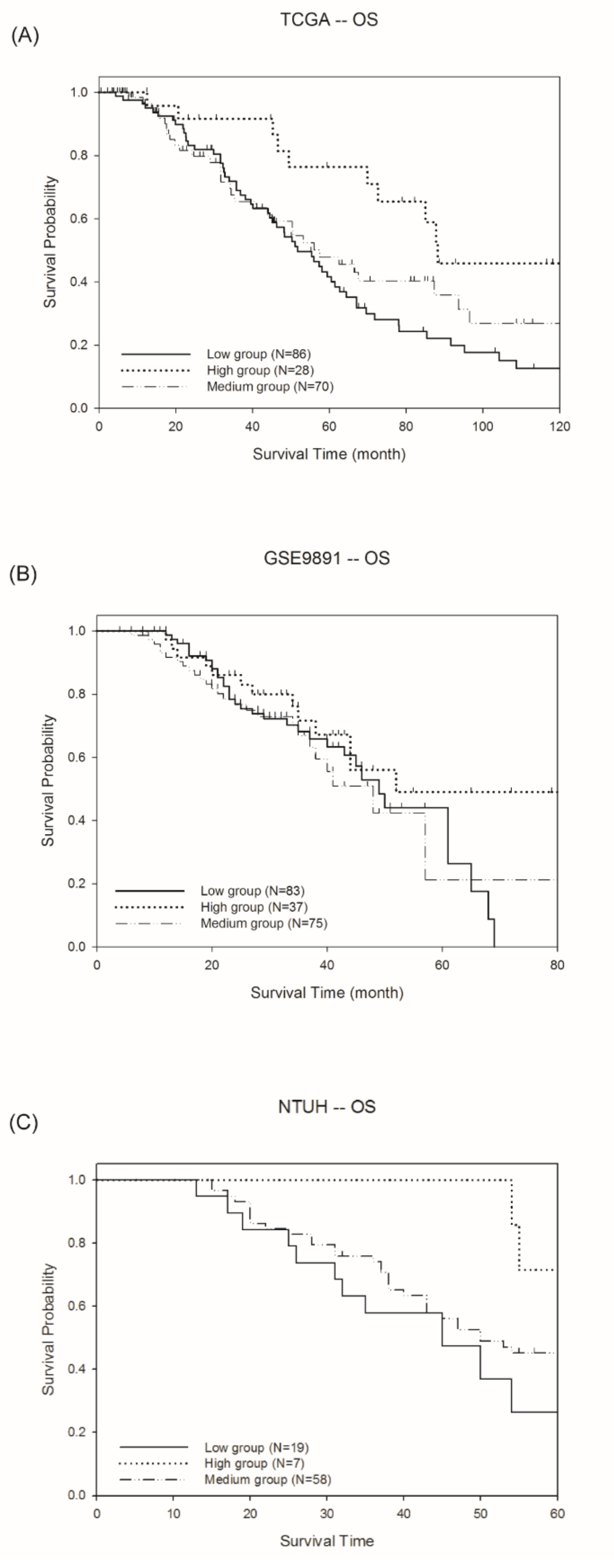

Figure S3. The Kaplan Meier survival curves of the OS in the three response groups in the (**A**) TCGA, (**B**) GSE9891 and (**C**) NTUH datasets.

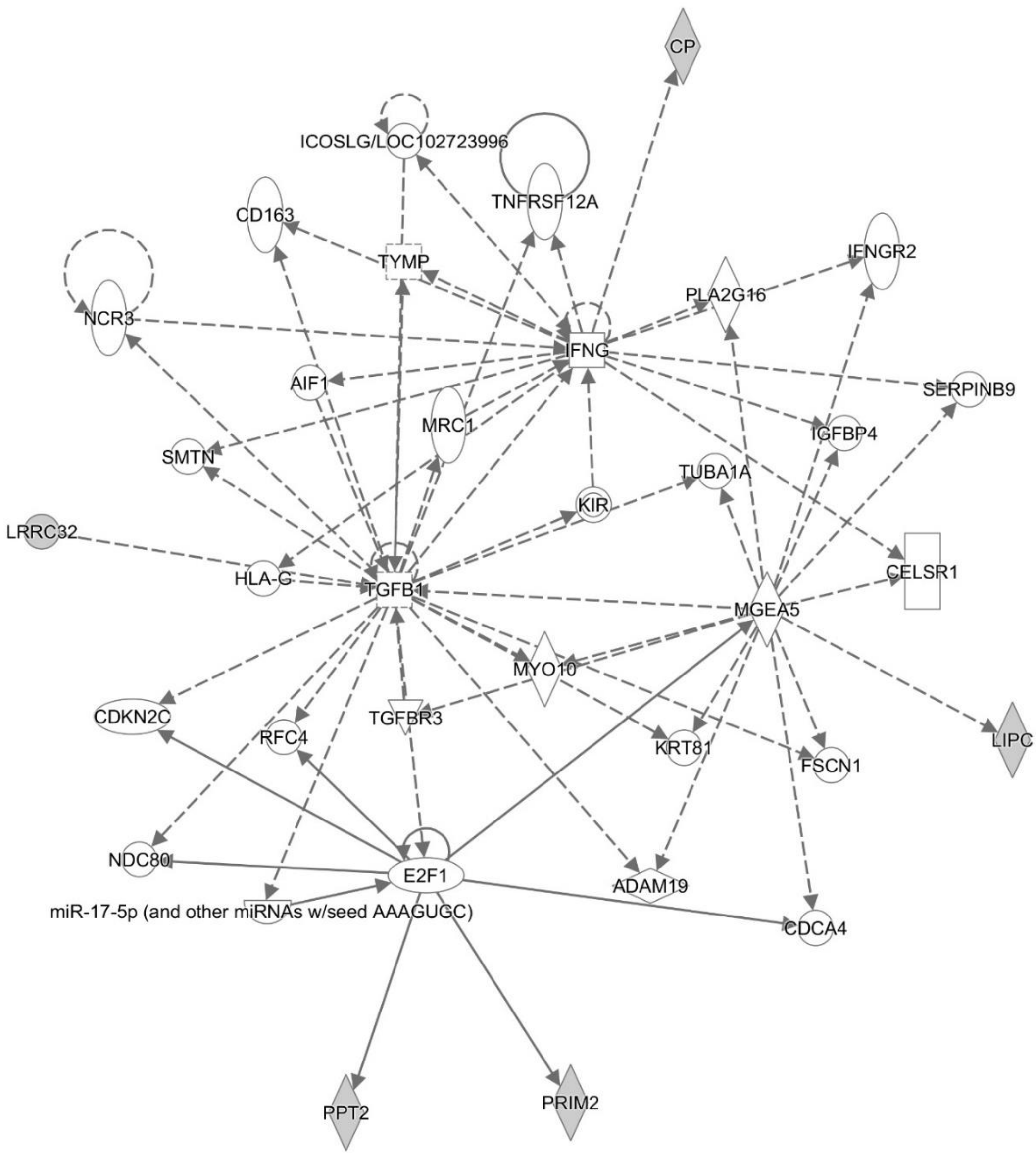

Figure S4. The gene-gene interaction network of the 10 genes analyzed using the Ingenuity Pathway Analysis website.

References

1. Barretina, J.; Caponigro, G.; Stransky, N.; Venkatesan, K.; Margolin, A.A.; Kim, S.; Wilson, C.J.; Lehar, J.; Kryukov, G.V.; Sonkin, D.; et al. The Cancer Cell Line Encyclopedia enables predictive modelling of anticancer drug sensitivity. *Nature* **2012**, *483*, 603–607, doi:10.1038/nature11003.
2. Edgar, R.; Domrachev, M.; Lash, A.E. Gene Expression Omnibus: NCBI gene expression and hybridization array data repository. *Nucleic Acids Res.* **2002**, *30*, 207–210.
3. Cancer Genome Atlas Research, N. Integrated genomic analyses of ovarian carcinoma. *Nature* **2011**, *474*, 609–615, doi:10.1038/nature10166.
4. Tothill, R.W.; Tinker, A.V.; George, J.; Brown, R.; Fox, S.B.; Lade, S.; Johnson, D.S.; Trivett, M.K.; Etemadmoghadam, D.; Locandro, B., et al. Novel molecular subtypes of serous and endometrioid ovarian cancer linked to clinical outcome. *Clin. Cancer Res.* **2008**, *14*, 5198–5208.

© 2019 by the authors. Submitted for possible open access publication under the terms and conditions of the Creative Commons Attribution (CC BY) license (http://creativecommons.org/licenses/by/4.0/).
